# Perception of obstetric violence and risk of post-traumatic stress disorder at 6 months postpartum: an observational study

**DOI:** 10.3389/fpsyg.2025.1712911

**Published:** 2025-12-16

**Authors:** Inmaculada Ortiz-Esquinas, Victoria Mazoteras-Pardo, Juan Miguel Martínez-Galiano, Ana Ballesta-Castillejos, Sandra Martínez-Rodríguez, Antonio Hernandez-Martinez

**Affiliations:** 1 Hospital Universitario Reina Sofía de Córdoba, Córdoba, Spain; 2 Instituto de Investigación Sanitaria de Castilla la Mancha (IDISCAM), Toledo, Spain; 3 Department of Nursing, Faculty of Nursing of Ciudad Real, University of Castilla-La Mancha, Ciudad Real, Spain; 4 Department of Nursing, University of Jaén, Jaén, Spain; 5 CIBER de Epidemiología y Salud Pública (CIBERESP), Madrid, Spain; 6 Department of Nursing, Faculty of Nursing of Albacete, University of Castilla-La Mancha, Albacete, Spain

**Keywords:** violence, obstetric, post-traumatic stress disorder, postpartum period, social support, mental health

## Abstract

**Introduction:**

Childbirth, traditionally viewed as a natural and positive process, can become a traumatic experience when obstetric violence or disrespectful treatment occurs. This type of experience can cause symptoms consistent with Post-Traumatic Stress Disorder negatively affecting maternal mental health, bonding with the newborn, and the development of the newborn.

**Objectives:**

To analyze the relationship between perceptions of inadequate treatment during childbirth and the risk of postpartum Post-Traumatic Stress Disorder at 6 months in a sample of women assessed 3 months after birth.

**Methods:**

An observational study with six-month follow-up was conducted in 341 women in Spain, initially recruited 3 months postpartum. Validated questionnaires were used: Childbirth Abuse and Respect Evaluation-Maternal Questionnaire (perceived abuse or disrespect), Perinatal Post Traumatic Stress Disorder, Family Apgar, and MOS social support survey Bivariate and multivariate analyses were performed using logistic regression.

**Results:**

Three hundred forty-one women participated, with a mean age of 33.38 years (SD = 4.23). 10.9% of the participants were at risk of Post-Traumatic Stress Disorder. Childbirth Abuse and Respect Evaluation-Maternal Questionnaire dimensions correlated positively with the Perinatal Post Traumatic Stress Disorder, with “inappropriate treatment by professionals” being the most significant (*r* = 0.60; 95% CI: 0.53–0.67). A greater perception of obstetric violence (Abuse and Respect Evaluation-Maternal Questionnaire ≥ P95) significantly increased the likelihood of developing Perinatal Post Traumatic Stress Disorder (aOR: 48.38; 95%CI: 10.07–232.44). Associations with risk of developing Post-Traumatic Stress Disorder were also observed for instrumental birth (aOR: 5.29; 95% CI: 1.53–18.28) and previous cesarean section (aOR: 7.54; 95% CI: 1.10–51.79). More social support was associated with a lower risk of Post-Traumatic Stress Disorder (aOR: 0.96; 95%CI: 0.94–0.99).

**Discussion and conclusion:**

A higher perception of obstetric violence is associated with an increased risk of developing postpartum Post-Traumatic Stress Disorder. Furthermore, invasive interventions such as instrumental births or previous cesarean sections increase psychological vulnerability. In contrast, social support acts as a protective factor. It is recommended to implement screening tools such as Abuse and Respect Evaluation-Maternal Questionnaire, reinforce training in respectful treatment, and promote humane care models to ensure the physical and emotional safety of women.

## Introduction

The humanization of childbirth—through respectful care, emotional support, and effective communication—is associated with improved maternal mental health, fostering a sense of control, safety, and well-being, and reducing the risk of postpartum post-traumatic stress and depression (Leinweber and Stramrood, 2024). Conversely, depersonalized or disrespectful care increases the likelihood of traumatic perceptions of childbirth, negatively affecting women’s emotional well-being (Ramirez-Perdomo et al., 2024; Yalley et al., 2024).

These findings are highly relevant, as recent studies indicate that a significant proportion of women, up to 34%, experience it as an emotionally disturbing event, and between 4 and 16% develop Post-traumatic Stress Disorder (PTSD) within the first year after birth (Ayers et al., 2016; Yildiz et al., 2017). According to DSM-5-TR criteria, this disorder is characterized by intrusive memories, avoidance behaviors, neurovegetative hyperarousal, and alterations in cognitive status or mood. Its persistence can lead to serious consequences, such as functional difficulties, impaired mother–child bonding, and negative effects on children’s emotional and cognitive development (Garthus-Niegel et al., 2017). The 6 months after childbirth are considered particularly sensitive for identifying these symptoms, as they coincide with the end of the initial adaptation phase and allow for the observation of clinical trajectories that may become chronic (Ayers et al., 2016).

The onset of postpartum PTSD is not always directly related to clinical complications, but in many cases, appears to be more closely linked to the perception of having experienced obstetric violence and/or disrespectful treatment during childbirth (Reed et al., 2017; Martinez-Vázquez et al., 2021; Ortiz-Esquinas et al., 2025). The World Health Organization (WHO) has defined obstetric violence as the appropriation of bodies and reproductive processes by healthcare personnel, which constitutes a violation of human rights. This violence can take multiple forms. On a physical level, it includes interventions such as repeated vaginal examinations without consent, systematic episiotomies, or the use of the Kristeller maneuver (Bohren et al., 2015; WHO, n.d.). On a psychological level, it manifests itself in contemptuous expressions, threats, or blaming (Savage and Castro, 2017) as well as unjustified restrictions, a lack of clear information, or not allowing the woman to be accompanied during labor (Vedam et al., 2019).

In an environment characterized by the inequality of power and vulnerability inherent in the biomedical model, what should be a physiological process becomes an experience of loss of control over one’s own body and a source of avoidable suffering (Diaz-Tello, 2016).

Three aspects of obstetric violence have been highlighted as particularly traumatic. The first is the failure to provide informed consent for procedures such as artificial rupture of membranes or episiotomy (Bohren et al., 2015). The second is emotional neglect by staff, which may include inattention or derogatory comments when faced with expressions of pain (Vedam et al., 2019). The third is medical coercion, which involves undue pressure to accept interventions such as unjustified cesarean sections or unnecessary neonatal separations (Savage and Castro, 2017).

From a neuronal and psychological perspective, these experiences generate fragmented traumatic memories that tend to be reactivated in the presence of childbirth-related stimuli, such as hospital smells or baby sounds, perpetuating the symptomatic cycle (Fenech and Thomson, 2014). Evaluating women after childbirth facilitates the detection of persistent PTSD, as one-third of acute cases progress to chronicity if left untreated (Yildiz et al., 2017). Furthermore, this stage marks a crucial transition toward full motherhood, so the functional impact of the disorder may intensify (Garthus-Niegel et al., 2017) and allows for a more precise distinction between common depressive symptoms and traumatic symptoms (Ayers et al., 2016).

Despite the growing visibility of the problem, significant research gaps remain. Most studies address the phenomenon in the first 3 months postpartum, which may underestimate the true prevalence of PTSD. Given the adverse consequences that postpartum PTSD can have on women and their families, it is essential to understand and address its potential relationship with experiences of inadequate treatment during childbirth. Identifying associated risk factors would allow for the establishment of care lines and clinical guidelines aimed at promoting safe and respectful environments during childbirth, with a positive impact on maternal mental health in the postnatal period.

## Methods

### Design and participants

A cross-sectional observational study was conducted at 6 months of follow-up in a sample of women recruited at 3 months postpartum, and the results have been published (Ortiz-Esquinas et al., 2025). To gather the required information, an online questionnaire was disseminated across associations related to pregnancy, childbirth, and the postpartum period, as well as breastfeeding support groups throughout Spain. Birth took place between June 2022 and December 2023 in Spain. The research received approval from the clinical research ethics committees of several hospitals. All participants were informed in writing about the study objectives and provided written informed consent before their inclusion. Exclusion criteria included women under 18 years of age and those with language barriers, i.e., those who did not speak or understand Spanish.

To determine the sample size, the maximum modeling principle was applied, which stipulates the inclusion of at least 10 subjects for each independent variable (Peduzzi et al., 1996). Considering that the prevalence of risk for Post-traumatic Stress Disorder (PTSD) in this population could reach up to 10% (Hernández-Martínez et al., 2021), it was estimated that 100 women at risk for PTSD were necessary to enable a multivariate analysis with a minimum of 10 independent variables.

### Data collection

Information was collected through an online questionnaire. The questionnaire covered several categories of variables, including sociodemographic data, obstetric history, details of the most recent birth, obstetric practices used, and neonatal outcomes, as well as the Childbirth Abuse and Respect Assessment Questionnaire - Maternal Version (CARE-MQ). This scale is composed of Likert-type questions that address various situations and practices related to obstetric violence (abuse and disrespect during childbirth). Response options range from “It did not occur during my birth” (0 points) to “It occurred and affected me a lot” (3 points), to “It occurred, but it did not affect me” (1 point), to “It occurred and affected me a little” (2 points). The total score ranges from 0 to 60 points. Scores can be categorized according to their percentile distribution (≤50th percentile, 51st-75th percentile, 75th-90th percentile, >90th percentile). The tool has demonstrated adequate internal consistency and excellent temporal stability in test–retest tests (Hernández-Martínez et al., 2024).

This information was already collected from the previous study. To recruit the participating women, we contacted each of the women who participated in the previous study and had expressed interest in continuing to participate in a subsequent follow-up via email. They were again informed and gave their consent to participate, providing a contact phone number or email address to address any questions they may have about the study.

The new questionnaire administered at the 6-month follow-up incorporated the following assessment tools:

- Perinatal Post-traumatic Stress Disorder Scale (PPQ) PTSD risk was assessed using this 14-item Likert-type questionnaire, with total scores ranging from 0 to 56 points (Hernández-Martínez et al., 2021). For this study, high risk for PTSD was defined as a score equal to or greater than the 90th percentile of its distribution.
- Family Apgar. The family Apgar assesses an individual’s perceived satisfaction with five dimensions of family dynamics: Adaptability, Partnership, Growth, Affection, and Resolve (Gómez Clavelina and Ponce Rosas, 2010). The scale comprises five items with three possible Likert-type responses: “Almost never” (0 points), “sometimes” (1 point), and “almost always” (2 points). The total score ranges from 0 to 10 points:

- 0–3 points. Indicates low satisfaction or a dysfunctional family.
- 4–6 points. Indicates medium satisfaction or possible dysfunction.
- 7–10 points indicate high satisfaction in a functional family.
  This scale has been validated in the Spanish population, showing good internal consistency (Cronbach’s *α* from 0.84 to 0.86), as well as adequate construct validity.
Medical Outcomes Study Social Support Survey (MOS-SSS) The MOS-SSS scale is a self-administered instrument designed to assess perceived social support. It consists of 19 items that explore various dimensions of social support, including emotional, instrumental, and affective support, as well as positive social interaction. The full version of the scale has been validated in the Spanish population through a descriptive study with a sample of 903 patients. The results showed excellent internal reliability (Cronbach’s *α* = 0.96), as well as a consistent unidimensional structure confirmed by exploratory and confirmatory factor analysis (KMO = 0.904; CFI = 0.95; TLI = 0.97; SRMR = 0.05) (Gómez-Campelo et al., 2014).

### Statistical analysis

Initially, a descriptive analysis of the data was conducted. Qualitative variables were described using absolute and relative frequencies, while the mean and standard deviation (SD) were calculated for quantitative variables.

Subsequently, the bivariate relationship between the overall CARE-MQ scale (first cutoff) and its dimensions and the overall PPQ scores (at 6 months) was analyzed using Pearson’s correlation coefficient. The next step was to identify the specific aspects of the CARE-MQ that were associated with a higher average PTSD risk score on the PPQ. This was done through an analysis of variance (ANOVA), examining the relationship of each CARE-MQ item with PPQ scores.

Finally, the relationship between sociodemographic, clinical, and birth experience factors and PTSD risk (defined by a PPQ score ≥19 points) was analyzed. All potential confounders were incorporated into this multivariate analysis. Crude and adjusted odds ratios (aORs), along with their respective 95% confidence intervals (95%CIs), were calculated using a binary logistic regression model (Backward Stepwise Regression). All analyses were performed using the statistical program SPSS 29.0.

## Results

### Sample characteristics

The study initially included 2,912 women who had given birth in the past 18 months. Of these, 479 met the eligibility criteria for having given birth within the first 3 months of the reference period and were invited to participate in the follow-up. In total, 341 women participated, representing a response rate of 71.3%.

The mean age was 33.38 years (SD: 4.23). 91.5% (312) of the pregnancies were planned. The majority of pregnancies were full-term (93.8%, 320), and singleton pregnancies predominated (99.4%, 339). The majority of participants were primiparous (80.1%, 273). Labor was induced in 43.7% (149) of the cases. Oxytocin was used to stimulate labor in 55.7% (190). Regional analgesia was used by 73.6% (251) of the women. Regarding skin-to-skin contact after birth, 62.8% (214) used it for at least 120 min.

Regarding PTSD risk at 6 months, the mean PPQ score was 12.46 (SD = 12.88), with 10.9% (Ford and Ayers, 2011) of women at risk for postpartum PTSD (defined as PPQ > 90th percentile). Meanwhile, the mean CARE-MQ score was 8.27 (SD = 11.56), with 7.6% (Beck and Watson, 2008) of women having a percentile ≥95th (≥31 points). The remaining characteristics of the sample are shown in Table 1.

**Table 1:** Sample characteristics.

Variable	*N* (%) *N* = 341	Mean (DE)
CARE- MQ		8.27 (11.56)
PPQ		12.46 (12.88)
Age		33.38 (4.23)
<2000 euros	57 (16.7)	
2000–3,000 euros	110(32.3)	
>3,000 euros	174 (51.0)	
Planned pregnancy		
No	29 (8.5)	
Yes	312 (91.5)	
Live birth		
No	3 (0.5)	
Yes	338 (99.1)	
Gestational age		
Term	320 (93.8)	
Premature	21 (6.2)	
Type of gestation		
Single	339 (99.4)	
Multiple	2 (0.6)	
Missing		
Previous cesarean section		
No	251 (73.6)	
Yes	90 (26.4)	
Number of pregnancies		
One	212 (62.2)	
Two	100 (29.3)	
Three or more	29 (8.5)	
Number of vaginal births		
None	74 (21.7)	
One	220 (64.5)	
Two or more	47 (13.8)	
Number of abortions		
None	242 (71)	
One	81 (23.8)	
Two or more	18 (5.3)	
Hypertension		
No	313 (91.8)	
Yes	28 (8.2)	
Diabetes		
No	311 (91.2)	
Yes	30 (8.8)	
APP		
No	323 (94.7)	
Yes	18 (5.3)	
Use of reproductive techniques		
No	290 (85)	
Yes	51 (15)	
Parity		
Primiparous	273 (80.1)	
Multiparous	68 (19.9)	
Antenatal education		
No	49 (14.4)	
Yes, but less than 5 classes	67 (19.6)	
Yes, at least 5 classes	225 (66)	
Induction of labor		
No	192 (56.3)	
Yes	149 (43.7)	
Birth plan		
No	105 (30.8)	
Yes, but it was not respected	65 (19.1)	
Yes, and it was mostly respected	171 (50.1)	
Health problems during previous birth		
No	251 (73.6)	
Yes	90 (26.4)	
Use of Oxytocin		
No	151 (44.3)	
Yes	190 (55.7)	
Regional		
No	90 (26.4)	
Yes	251 (73.6)	
Nitrous oxide		
No	328 (96.2)	
Yes	13 (3.8)	
General		
No	325 (95.3)	
Yes	16 (4.7)	
Type of birth		
Normal	195 (57.2)	
Instrumental	65 (19.1)	
Planned cesarean	11 (3.2)	
Emergency cesarean	70 (20.5)	
Episiotomy		
No	273 (80.1)	
Yes	68 (19.9)	
Severe tear		
No	325 (95.3)	
Yes	16 (4.7)	
Skin-to-skin following birth for at least 120 min		
No	127 (37.2)	
Yes	214 (62.8)	
Breastfeeding in the first hour		
No	90 (26.4)	
Yes	251 (73.6)	
Neonatal admission		
No	288 (84.5)	
Yes	53 (15.5)	
Maternal ICU admission		
No	337 (98.8)	
Yes	4 (1.2)	
Maternal readmission following discharge		
No	331 (97.1)	
Yes	10 (2.9)	
Surgical intervention following birth		
No	316 (92.7)	
Yes	25 (7.3)	
CARE- MQ Score		8.27 (11.56)
CARE-MQ (grouped by percentiles)		
Percentile <50 (3 points)	169 (49.6)	
Percentile 51–75 (3–11 points)	80 (23.5)	
Percentile 76–90 (11–22 points)	42 (12.3)	
Percentile 91–94 (22–31 points)	24 (7.0)	
*p* > 95 (> = 31 points)	26 (7.6)	
PPQ score		12.46 (12.88)
PTSD (PPQ>90 percentile)		
No	304 (89.1)	
Yes	37 (10.9)	
Apgar familiar score		15.2 (4.57)
Social Support Score (MOS scale)		82.1 (14.46)

### Correlation between the CARE-MQ dimensions and PPQ scores at 6-month follow-up

**Figure 1:**
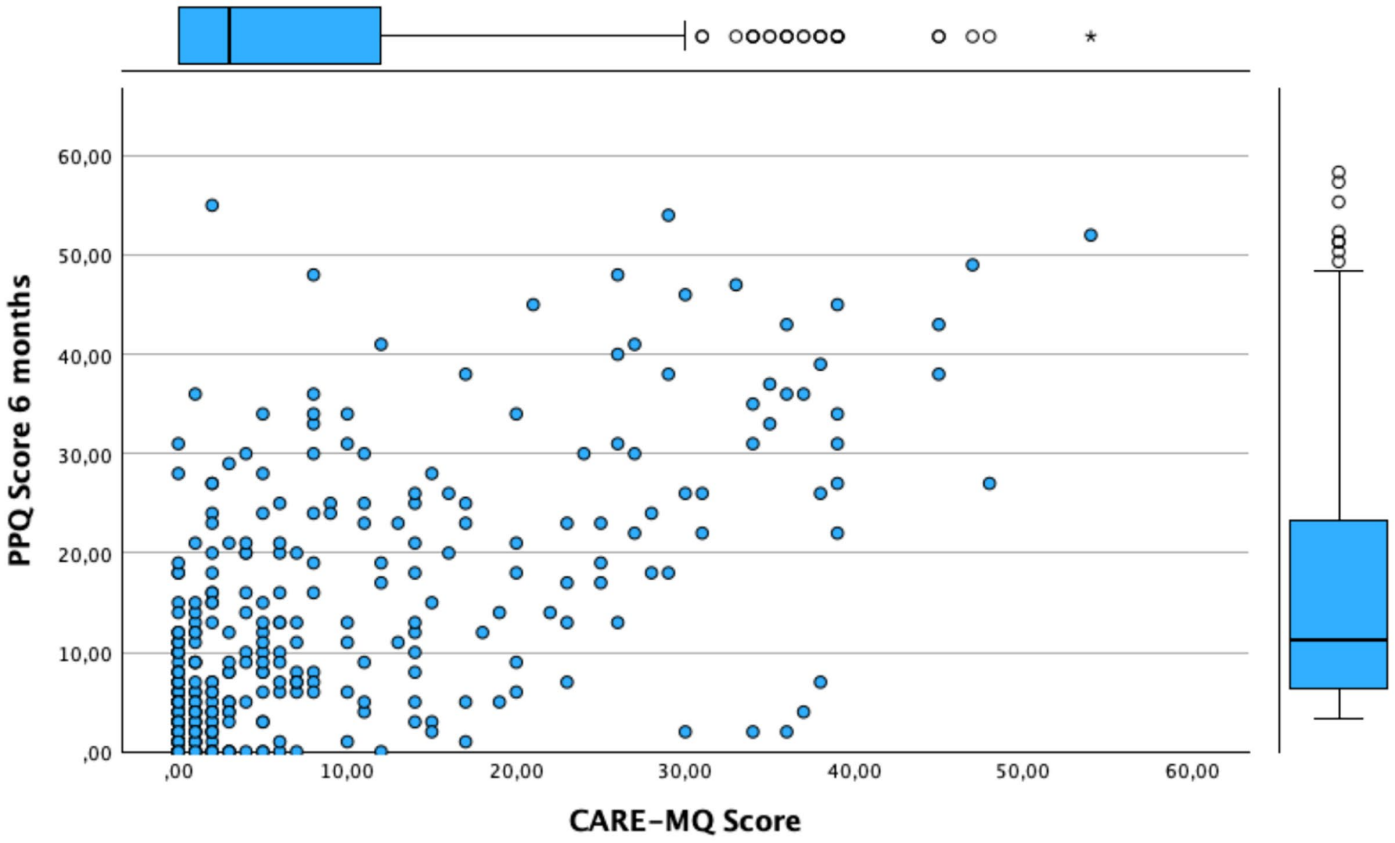
Relationship between CARE-MQ and PPQ scores at 6-month follow-up.

The correlation between the CARE-MQ dimensions and PPQ scores was analyzed, yielding a positive and statistically significant correlation across all dimensions and overall: “Emotional abuse” with *r* = 0.55 (95% CI: 0.47–0.62), “inadequate treatment by professionals” with *r* = 0.60 (95% CI: 0.53–0.67), “physical abuse” *r* = 0.42 (95% CI: 0.33–0.50), and “separation” *r* = 0.42 (95% CI: 0.33–0.51), which correlated positively (*p* < 0.001) with PTSD at 6 months. Inadequate treatment by professionals was the element that correlated the most. The correlation between the CARE-MQ score and PPQ at 6 months was 0.63 (95%CI: 0.56–0.69). This relationship is illustrated graphically in Figure 1.

### Correlation between the CARE-MQ scores and PPQ scores at 6-month follow-up

Next, to analyze the differences in scores between the CARE-MQ scale and the PPQ scores, an analysis of variance (ANOVA) was performed, revealing a statistically significant linear trend for all items (*p* < 0.001).

As can be seen in Table 2, there is a significant linear trend (*p* < 0.001): the greater the perception of negative experiences (from lack of information to physical violence), the higher the PPQ scores and, therefore, the risk of PTSD. The most striking aspects were physical violence, lack of professional support, and lack of clear information.

**Table 2:** Relationship between the dimensions of the CARE-MQ questionnaire and the PPQ questionnaire scores.

CARE-MQ questionnaire	(0 points) Mean (SD)	(1 point) Mean (SD)	(2 points) Mean (SD)	(3 points) Mean (SD)	*P-*value
Items on information received from the professionals (Items 1–3)					
	Information received	Information not given, but it did not affect me AT ALL	Information not given, and it affected me A LITTLE	Information not given, and it affected me A LOT	
1. The professionals that assisted at my birth introduced themselves by name and profession.	9.39 (9.75)	13.67 (13.22)	20.25 (15.35)	32.00 (12.35)	**<0.001**
	It occurred	It did NOT occur, but it did not affect me AT ALL	It did NOT occur, and affected me A LITTLE	It did NOT occur, and affected me A LOT	
2. They explained to me the techniques and/or procedures that were going to be performed on me (for example, placing an IV, rupturing the amniotic sac, administering medication, etc.) and the reason why, the alternatives, as well as the risks and benefits of them in an understandable way, and/or I was able to ask the questions that arose and choose between the proposed alternatives.	9.07 (9.89)	18.21 (13.99)	16.71 (13.13)	26.71 (14.47)	**<0.001**
	It occurred	It did NOT occur, but it did not affect me AT ALL	It did NOT occur, and affected me A LITTLE	It did NOT occur, and affected me A LOT	
3. They explained clearly how my labor was progressing, or my health status, or that of my infant, in a way that I could understand and/or I was able to ask any questions I had.	8.53 (9.14)	15.30 (10.55)	18.65 (13.66)	27.50 (14.33)	**<0.001**
Items regarding privacy (Items 4–5)					
	It occurred	It did NOT occur, but it did not affect me AT ALL	It did NOT occur, and affected me A LITTLE	It did NOT occur, and affected me A LOT	
4. The professionals who treated me protected my privacy (using screens, covering my private parts, etc.)	9.74 (10.22)	14.09 (12.26)	23.58 (15.46)	29.53 (14.92)	**<0.001**
	It did NOT occur	It occurred, but it did NOT affect me AT ALL	It occurred, and affected me A LITTLE	It occurred, and affected me A LOT	
5. During vaginal examinations and/or techniques, there were more people present than necessary (other doctors, nurses, orderlies, cleaning staff, etc.) or students (nursing, medicine) were present without anyone having asked my permission.	9.81 (10.89)	16.29 (14.09)	18.09 (11.38)	28.26 (14.99)	**<0.001**
Items regarding professional support and care received (6–9)					
	They allowed me	They did not allow me, but it did not affect me AT ALL	They did not allow me, and it affected me A LITTLE	They did not allow me, and it affected me A LOT	
6. I was allowed to be accompanied by the person I chose during the entire birth process.	11.07 (11.81)	18.86 (9.58)	15.11 (11.27)	23.79 (14.81)	**<0.001**
	Yes, I was assisted	I was not assisted, but it did not affect me AT ALL	I was not assisted, and affected me A LITTLE	I was not assisted, and affected me A LOT	
7. When I requested help (to move, wash myself, pain relief, etc.) I was NOT assisted.	9.82 (10.45)	21.43 (13.29)	20.29 (11.99)	35.24 (10.43)	**<0.001**
	They helped me and answered my questions	They did not help me, but it did not affect me AT ALL	They did not help me, and it affected me A LITTLE	They did not help me, and it affected me A LOT	
8. I was helped with care of my newborn, breastfeeding or artificial feeding, and they did NOT answer my questions.	10.24 (0.7)	14.75 (12.52)	14.68 (13.22)	22.45 (15.59)	**<0.001**
	Yes, they respected it	They did not respect it, but it did not affect me AT ALL	They did not respect it, and it affected me A LITTLE	They did not respect it, and it affected me A LOT	
9. The professionals respected my birth plan when possible and when not possible they explained the reason to me and we agreed on an alternative.	8.74 (9.59)	19.69 (7.89)	17.73 (13.44)	27.96 (14.11)	**<0.001**
Items regarding inadequate interpersonal relationship (Items 10–14)					
	It did NOT occur	It occurred, but it did NOT affect me AT ALL	It occurred, and affected me A LITTLE	It occurred, and affected me A LOT	
10. I was told off during childbirth or my questions and doubts were answered disrespectfully (with criticism, yelling, or abuse).	10.22 (10.92)	19.00 (10.94)	17.83 (14.96)	29.61 (13.02)	**<0.001**
	It did NOT occur	It occurred, but it did NOT affect me AT ALL	It occurred, and affected me A LITTLE	It occurred, and affected me A LOT	
11. They verbally scared or intimidated me about a danger to me or my baby into accepting certain practices that I did not agree with and they did NOT explain to me why they carried them out or with what justification.	9.58 (10.38)	17.82 (7.86)	19.74 (12.92)	28.83 (14.43)	**<0.001**
	It did NOT occur	It occurred, but it did NOT affect me AT ALL	It occurred, and affected me A LITTLE	It occurred, and affected me A LOT	
12. They spoke to me like I was a child or mocked me.	10.62 (11.29)	15.77 (9.21)	17.93 (15.34)	27.00 (14.79)	**<0.001**
	It did NOT occur	It occurred, but it did NOT affect me AT ALL	It occurred, and affected me A LITTLE	It occurred, and affected me A LOT	
13. I was criticized during childbirth for expressing my emotions (crying, yelling in pain, etc.)	10.83 (11.48)	15.82 (11.41)	17.94 (13.08)	29.47 (14.42)	**<0.001**
	It did NOT occur	It occurred, but it did NOT affect me AT ALL	It occurred, and affected me A LITTLE	It occurred, and affected me A LOT	
14. During the birth experience, I was made to feel vulnerable, guilty, insecure, or that I had not lived up to what was expected of me (that I had not collaborated).	9.83 (10.49)	20.91 (7.38)	10.73 (16.86)	29.83 (12.99)	**<0.001**
Items on inadequate or innecessary procedures (Items 15–20)					
	They allowed me	They did not allow me, but it did not affect me AT ALL	They did not allow me, and it affected me A LITTLE	They did not allow me, and it affected me A LOT	
15. They allowed me to adopt the position that I requested during dilation and delivery when not contraindicated.	10.09 (11.06)	16.38 (9.53)	16.39 (11.23)	31.08 (14.59)	**<0.001**
	Yes, they used it	They did not use it, but it did not affect me AT ALL	They did not use it, and it affected me A LITTLE	They did not use it, and it affected me A LOT	
16. They used anesthesia, whether requested or not, for example, to suture a tear or episiotomy or manually remove the placenta.	11.83 (12.15)	15.5 (12.83)	21.00 (8.89)	41.00 (8.83)	**<0.001**
	Yes, they used measures	They did not use it, but it did not affect me AT ALL	They did not use it, and it affected me A LITTLE	They did not use it, and it affected me A LOT	
17. The vaginal examinations were performed on me without taking measures to reduce the discomfort that this entails (use of lubricant, performing the technique progressively, trying to relax)	10.38 (11.49)	15.48 (11.66)	18.06 (12.31)	24.69 (15.47)	**<0.001**
	Yes, with my consent	It occurred without my consent, but it did not affect me AT ALL	It occurred without my consent, and it affected me A LITTLE	It occurred without my consent, and it affected me A LOT	
18. They carried out some of these practices without my consent (enema, shaving, vaginal examinations, episiotomy, abdominal pressure).	10.21 (11.03)	14.32 (10.27)	17.08 (13.6)	26.72 (15.47)	**<0.001**
	It did NOT occur	It occurred, but it did NOT affect me AT ALL	It occurred, and affected me A LITTLE	It occurred, and affected me A LOT	
19. I experienced some type of physical violence during labor. For example, I was slapped on the face or slapped on the thighs during childbirth to scold me or reprimand me for my behavior.	11.57 (11.76)	26.25 (13.19)	46.5 (2.12)	42.0 (8.72)	**<0.001**
	They allowed it/offered it	They did not allow it/offer it, but it did not affect me AT ALL	They did not allow me/, and it affected me A LITTLE	They did not allow it/ offer it, but it did not affect me A LOT	
20a. I was allowed to do skin-to-skin immediately after giving birth without reasons and/or without giving explanations that would contraindicate it. (Only women with a live birth) 20b. They offered me the possibility of seeing my baby or preparing a memory box. (Only women with fetal loss)	10.09 (10.95)	13.25 (12.82)	19.95 (11.79)	26.25 (14.24)	**<0.001**

### Risk of post-traumatic stress

**Figure 2:**
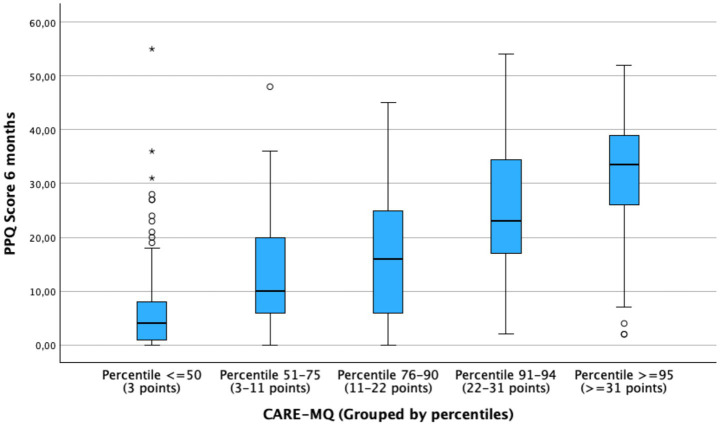
Relationship between CARE-MQ scores and PPQ scores grouped by percentiles to detect risk for post-traumatic stress disorder.

A bivariate analysis was then performed to identify the relationship between various sociodemographic and clinical factors and the risk of postpartum PTSD. As shown in Table 3, a significant association was observed between high scores on the CARE-MQ scale (Figure 2), grouped by percentiles, and an increased risk of developing PTSD. The same was true for women with: previous cesarean section, induced labor, birth plans being disrespected, complicated births, use of regional analgesia, instrumental births, scheduled cesarean section, emergency cesarean section, episiotomy, and hospitalization of the newborn. In contrast, women who had more vaginal births, had skin-to-skin contact for at least 120 min, breastfed within the first hour, and had higher scores on the Family and Social Support (MOS) Apgar score, had lower scores for PTSD.

**Table 3:** Factors associated with the risk of PTSD Bivariate and multivariate analysis.

Variables	PTSD (PPQ>90 percentile)		Bivariate analysis		Multivariate analysis	
	No *n* (%) *N* = 304	Yes *n* (%) *N* = 37	OR 95% CI	*p*-value	aOR 95% CI	*p*-value
CARE– MQ				**<0.001**		**<0.001**
Percentile <50 (3 points)	166 (98.2)	3 (1.8)	1		1	
Percentile 51–75 (3–11 points)	73 (91.3)	7 (8.8)	**5.30 (1.33–21.09)**	**0.018**	3.09 (0.70–13.68)	0.138
Percentile 76–90 (11–22 points)	38 (90.5)	4 (9.5)	**5.83 (1.25–27.11)**	**0.025**	2.53 (0.46–13.79)	0.284
Percentile 91–94 (22–31 points)	17 (70.8)	7 (29.2)	**22.78 (5.38–96.32)**	**<0.001**	**12.33 (2.37–64.28)**	**0.003**
*P* > 95 (> = 31 points)	10 (38.5)	16 (61.5)	**88.53 (22.08–354.90)**	**<0.001**	**48.38 (10.07–232.44)**	**<0.001**
Maternal age mean (SD)	33.8 (4.25)	34.5 (4.03)	1.04 (0.96–1.12)	0.335		
Family monthly income in euros				0.605		
<2000 euros	50 (87.7)	7 (12.3)	1			
2000–3,000 euros	96 (87.3)	14 (12.7)	1.04 (0.40–2.75)	0.934		
>3,000 euros	158 (90.8)	16 (9.2)	0.72 (0.28–1.86)	0.501		
Planned pregnancy				0.596		
No	25 (86.2)	4 (13.8)	1			
Yes	279 (89.4)	33 (10.6)	0.73 (0.24–2.25)			
Live birth				0.999		
No	3 (100)	0 (0.0)	1			
Yes	301 (89.1)	37 (10.9)	N/A			
Gestational age				0.922		
Term	286 (89.4)	34 (10.6)	1			
Premature	18 (85.7)	3 (14.3)	1.57 (0.43–5.69)	0.487		
Type of gestation				0.135		
Single	303 (89.4)	36 (10.6)	1			
Multiple	1 (50.0)	1 (50.0)	8.41 (0.51–137.47)			
Previous cesareans				**<0.001**		0.039
No	235 (93.6)	16 (6.4)	1		1	
Yes	69 (76.7)	21 (23.3)	**4.89 (2.41–9.93)**	**<0.001**	**7.55 (1.10–51.71)**	
Number of pregnancies				0.937		
One	188 (88.7)	24 (11.3)	1			
Two	90 (90.0)	10 (10.0)	0.87 (0.39–1.89)	0.727		
Three or more	26 (89.7)	3 (10.3)	0.90 (0.25–3.21)	0.876		
Number of vaginal births				0.010		
None	59 (79.7)	15 (20.3)	1			
One	199 (90.5)	21 (9.5)	**0.41 (0.20–0.85)**	**0.017**		
Two or more	46 (97.9)	1 (2.1)	**0.08 (0.01–0.67)**	**0.019**		
Number of abortions				0.769		
None	215 (88.8)	27 (11.2)	1			
One	72 (88.9)	9 (11.11)	0.99 (0.44–2.21)	0.991		
Two or more	17 (94.4)	1 (5.6)	0.46 (0.06–3.66)	0.470		
Hypertension				0.225		
No	277 (88.5)	36 (11.5)	1			
Yes	27 (96.4)	1 (3.6)	0.28 (0.03–2.16)			
Diabetes				0.099		
No	280 (90.0)	31 (10.0)	1			
Yes	24 (80.0)	6 (20.0)	2.25 (0.85–5.94)			
Threatened preterm birth				0.998		
No	286 (88.5)	37 (11.5)	1			
Yes	18 (100)	0 (0.0)	N/A			
Use of fertility treatments				0.820		
No	259 (89.3)	31 (10.7)	1			
Yes	45 (88.2)	6 (11.8)	1.11 (0.44–2.82)			
Parity				0.002		
Primiparous	243 (89.0)	30 (11.0)	1			
Multiparous	61 (89.7)	7 (10.3)	0.59 (0.42–0.82)			
Antenatal education				0.946		
No	44 (89.8)	5 (10.2)	1			
Yes, but less than 5 classes	59 (88.1)	8 (11.9)	1.19 (0.36–3.89)	0.770		
Yes, at least 5 classes	201 (89.3)	24 (10.7)	1.05 (0.38–2.90)	0.924		
Induction of labor				**0.019**		
No	178 (92.7)	14 (7.3)	1			
Yes	126 (88.6)	23 (15.4)	**2.32 (1.15–4.68)**			
Birth plan				**<0.001**		
No	101 (96.2)	4 (3.8)	1			
Yes, but it was not respected	40 (61.5)	25 (38.5)	**15.78 (5.16–48.23)**	**<0.001**		
Yes, and it was mostly respected	163 (95.3)	8 (4.7)	1.23 (0.36–4.22)	0.732		
Problems during previous birth				**<0.001**		0.059
No	235 (93.6)	16 (6.4)	1		1	
Yes	69 (76.7)	21 (23.3)	**4.47 (2.21–9.03)**		2.46 (0.97–6.28)	
Use of oxytocin				0.128		
No	139 (92.1)	12 (7.9)	1			
Yes	165 (86.8)	25 (13.2)	1.75 (0.85–3.62)			
Use of regional anesthesia				**0.030**		
No	86 (95.6)	4 (4.4)	1			
Yes	218 (86.9)	33 (13.1)	**3.25 (1.11–9.46)**			
Use of nitrous oxide				0.595		
No	293 (89.3)	35 (10.7)	1			
Yes	11 (84.6)	2 (15.4)	1.52 (0.32–7.14)			
General anesthesia				0.307		
No	291 (89.5)	34 (10.5)	1			
Yes	13 (81.3)	3 (18.8)	1.97 (0.53–7.28)			
Type of birth				**<0.001**		**0.004**
Normal	190 (97.4)	5 (2.6)	1		1	
Instrumental	51 (78.5)	14 (21.5)	**10.43 (3.58–30.31)**	**<0.001**	**5.29 (1.53–18.28)**	**0.009**
Planned cesarean	6 (54.5)	5 (45.5)	**31.66 (7.19–139.42)**	**0.002**	2.41 (0.18–33.04)	0.510
Emergency cesarean	57 (81.4)	13 (18.6)	**8.66 (2.96–25.34)**	**<0.001**	0.40 (0.05–3.53)	0.410
Episiotomy				**0.048**		
No	248 (90.8)	25 (9.2)	1			
Yes	56 (82.4)	12 (17.6)	**2.12 (1.01–4.48)**			
Severe tear				0.074		
No	292 (89.8)	33 (10.2)	1			
Yes	12 (75.0)	4 (25.0)	2.94 (0.9–9.67)	0.074		
Skin-to-skin for at least 120 min				**<0.001**		
No	102 (80.3)	25 (19.7)	1			
Yes	202 (94.4)	12 (5.6)	**0.24 (0.12–0.50)**	**<0.001**		
Breastfeeding in first hour				**0.016**		
No	74 (82.2)	16 (17.8)	1			
Yes	230 (91.6)	21 (8.4)	**0.42 (0.20–0.85)**			
Admission of newborn				**0.046**		
No	261 (90.6)	27 (9.4)	**1**			
Yes	43 (81.1)	10 (18.9)	**2.25 (1.02–4.97)**			
Maternal ICU admission				0.380		
No	301 (89.3)	36 (10.7)	1			
Yes	3 (75.0)	1 (25.0)	2.78 (0.28–27.50)			
Hospital readmission				0.356		
No	296 (89.4)	35 (10.8)	1			
Yes	8 (80.0)	2 (20.0)	2.11 (0.43–10.35)			
Postpartum surgical intervention				0.135		
No	284 (89.9)	32 (10.1)	1			
Yes	20 (80.0)	5 (20.0)	2.21 (0.78–6.31)	0.135		
Neonatal admission				**0.046**		
No	261 (90.6)	27 (9.4)	1			
Yes	43 (81.1)	10 (18.9)	**2.24 (1.01–4.97)**			
Social support_MOS Mean (SD)	83.0 (14.05)	75.1 (15.94)	**0.97 (0.95–0.99)**	**0.002**	**0.96 (0.94–0.99)**	**0.013**
Family Apgar familiar Mean (SD)	15.4 (4.41)	12.9 (5.32)	**0.90 (0.85–0.96)**	**0.002**		

SD: Standard deviation; CI: Confidence Interval; OR: Odds Ratio; aOR: Adjusted Odds Ratio; NC: Not calculated. Statistically significant values in bold.

Finally, a multivariate analysis was performed to control for confounding bias, observing that women with a higher perception of reported obstetric violence had a higher risk of PTSD, specifically the highest probability in the group with score percentiles ≥95, where the odds of PTSD were 48.38 times higher (aOR) (95% CI: 10.07–232.44) compared to those with CARE-MQ scores ≤P50. In this same analysis, it was observed that instrumental birth (aOR: 5.29; 95%CI: 1.53–18.28) and previous cesarean (aOR: 7.54; 95%CI: 1.10–51.79) were more likely to develop PTSD. In contrast, women with greater social support as assessed by the MOS questionnaire were less likely (aOR: 0.96; 95%CI: 0.94–0.99).

## Discussion

The results show that 10.9% of women were at risk for PTSD. This prevalence is in line with previous literature, which estimates a prevalence between 3 and 16% depending on the instrument used and the characteristics of the healthcare and sociocultural context (Grekin and O’Hara, 2014; Dekel et al., 2019; Yildiz et al., 2017).

One of the main contributions of this study is the significant association between scores on the CARE-MQ scale, which measures experiences of obstetric violence, and the risk of PTSD. As the impairment in the items on this scale increased, so did the score on the PPQ scale, reinforcing the importance of the perception of childbirth as a potential trigger for psychological trauma (Simpson et al., 2018; Ayers, 2004; Beck and Watson, 2008). This result is supported by existing literature, which has documented the negative impact of obstetric violence and dehumanizing treatment on maternal mental health after childbirth (Slade et al., 2019; Sen et al., 2018; Bohren et al., 2015). Likewise, recent research has shown that the subjective perception of abuse can generate psychological consequences even when the clinical outcomes of childbirth have been favorable (Bohren et al., 2015).

Particularly striking was the finding regarding items 19 and 16 of the CARE-MQ, which refer to physical aggression, showing the highest mean scores on the PPQ. This result points to the serious impact of experiences that women identify as aggressive or violent during childbirth, although these do not always correspond to situations objectively classified as obstetric violence. This phenomenon has been noted by other authors, who emphasize that the perception of abuse or loss of autonomy can have psychological consequences as important as adverse clinical events (Bohren et al., 2015).

Furthermore, multivariate analysis identified instrumental birth and previous cesarean section as risk factors, consistent with previous findings linking these events with feelings of invasion, loss of control, or lack of consent (Ford and Ayers, 2011; Harris and Ayers, 2012). These experiences can lead to a perception of loss of safety and autonomy in women during childbirth, which is a trigger for postpartum PTSD (Beck, 2004).

Interventions performed during instrumental births can be perceived as invasive experiences, intensifying the negative emotional impact of birth. Qualitative studies have described how women who experience instrumental births feel a lack of participation, reinforcing their sense of trauma (Fenech and Thomson, 2014; Söderquist et al., 2009). Similarly, multiple studies have found that non-eutocic births, especially when unexpected or performed without sufficient information or consent, increase the risk of PTSD (Czarnocka and Slade, 2000; Ford and Ayers, 2011; Fenech and Thomson, 2014).

Conversely, perceived social support, assessed using the MOS questionnaire, was associated with a lower likelihood of developing PTSD, which is consistent with studies demonstrating that an emotional support network acts as a key protective factor in potentially traumatic situations (Milgrom et al., 2019; Leight et al., 2010). Furthermore, it corroborates that emotional support contributes to a more positive birth experience by facilitating the validation of experienced emotions and reducing feelings of isolation or perceived failure that can arise after complicated births (Tani and Castagna, 2017).

Among the strengths of this study are the combined use of validated tools for assessing PTSD (PPQ) and negative experiences in care (CARE-MQ), as well as the multivariate analysis to control for confounding variables. However, some limitations should be noted. First, the observational design prevents the establishment of causal relationships. Second, the PTSD assessment was based on a screening scale and not a clinical diagnosis. A further limitation is the lack of statistical power due to the sample size, meaning that variables traditionally associated with a higher risk of PTSD were not included in our study.

The results support the need to implement systematic tools for assessing negative childbirth experiences, such as the CARE-MQ questionnaire, which could be integrated into postpartum care to identify women at risk and provide them with early psychological care. Furthermore, the study highlights the importance of training healthcare professionals in communication skills and in managing complex obstetric situations from a woman-centered perspective (Balde et al., 2020).

Moreover, the interpretation of these results must be contextualized within the Spanish cultural and healthcare model, which is characterized by predominantly hospital-based childbirth care and a high use of obstetric interventions compared with other European countries (Euro-Peristat Project, 2022). This environment may influence women’s perceptions, as highly medicalized practices may be experienced either as routine or, conversely, as invasive in the absence of adequate communication. Likewise, the growing social and regulatory debate on obstetric violence in Spain—driven by international organizations such as the WHO (World Health Organization, 2014) and by reproductive rights advocacy movements—may increase sensitivity toward experiences perceived as disrespectful. These cultural and systemic factors should be considered when interpreting the findings and when assessing their generalizability to contexts with less medicalized models of care or greater continuity of care (El Parto es Nuestro, 2020).

## Conclusion

This study highlights the relationship between the quality of obstetric care, negative experiences during childbirth, and the risk of postpartum post-traumatic stress disorder at 6-month follow-up. The results indicate that the greater the perception of obstetric violence, the greater the risk of developing postpartum PTSD. Inadequate treatment by providers was the CARE-MQ scale item most strongly correlated with the risk of PTSD. A significant and linear association exists between higher scores on the CARE-MQ scale items and higher PPQ scores at the 6-month follow-up. Risk factors identified for developing PTSD include instrumental birth and previous cesarean section, with greater social support being a protective factor for PTSD.

This evidence reinforces the need to move toward a more humanized, woman-centered model of care based on respect, autonomy, and empathy as key elements for safety, not only physical but also emotional and psychological.

## Data Availability

The raw data supporting the conclusions of this article will be made available by the authors, without undue reservation.
