# Correction: Perception of obstetric violence and risk of post-traumatic stress disorder at 6 months postpartum: an observational study

**DOI:** 10.3389/fpsyg.2026.1777503

**Published:** 2026-01-23

**Authors:** Inmaculada Ortiz-Esquinas, Victoria Mazoteras-Pardo, Juan Miguel Martínez-Galiano, Ana Ballesta-Castillejos, Sandra Martínez-Rodríguez, Antonio Hernandez-Martinez

**Affiliations:** 1 Hospital Universitario Reina Sofía de Córdoba, Córdoba, Spain; 2 Instituto de Investigación Sanitaria de Castilla la Mancha (IDISCAM), Toledo, Spain; 3 Department of Nursing, Faculty of Nursing of Ciudad Real, University of Castilla-La Mancha, Ciudad Real, Spain; 4 Department of Nursing, University of Jaén, Jaén, Spain; 5 CIBER de Epidemiología y Salud Pública (CIBERESP), Madrid, Spain; 6 Department of Nursing, Faculty of Nursing of Albacete, University of Castilla-La Mancha, Albacete, Spain

**Keywords:** violence, obstetric, post-traumatic stress disorder, postpartum period, social support, mental health

Affiliation 4 was erroneously typeset as

“Department of Health Sciences, University of Jaén, Jaén, Spain”.

The corrected affiliation appears below:

“Department of Nursing, University of Jaén, Jaén, Spain”.

The original version of this article has been updated.

